# Rare Case of Cavernous Haemangioma of the Right Atrium with Probable Hepatic Haemangioma

**DOI:** 10.1155/2022/9214196

**Published:** 2022-02-27

**Authors:** Sally Harrison

**Affiliations:** Dunedin Public Hospital, Dunedin, New Zealand

## Abstract

Cardiac haemangiomas are rare causes of atrial masses. This case report is of a 44-year-old male who presented with a right atrial mass that was found incidentally on a CT performed for renal colic. The mass was further investigated with a transthoracic echocardiogram that showed that it was echodense and arising from the Eustachian valve in the right atrium. Coronary angiogram revealed large well-developed atrial branches that crossed superiorly over the left atrium and entered the mass in the right atrium. Surgical resection was undertaken, and this confirmed that the mass had a fleshy, encapsulated appearance with a sessile stalk. Histology demonstrated a cavernous haemangioma. The patient had a residual small defect in the interatrial septum postoperatively but otherwise made a good recovery.

## 1. Introduction

Cardiac tumours are uncommon medical entities. 75% of primary cardiac tumours are benign in nature, with atrial myxomas being the most common of these, making up 75% [1].

The following case report is of a 44-year-old patient with a right atrial mass. This is an example of a mass which was suspicious for myxoma but with atypical features.

## 2. Case Presentation

A 44-year-old gentleman presented with an episode of right-sided renal colic and was referred for an US abdomen. On this imaging, an echogenic mass was incidentally seen within the right atrium. Due to the patient's ongoing abdominal discomfort, a CT abdomen with arterial and venous contrast was also performed.

**Figure 1:**
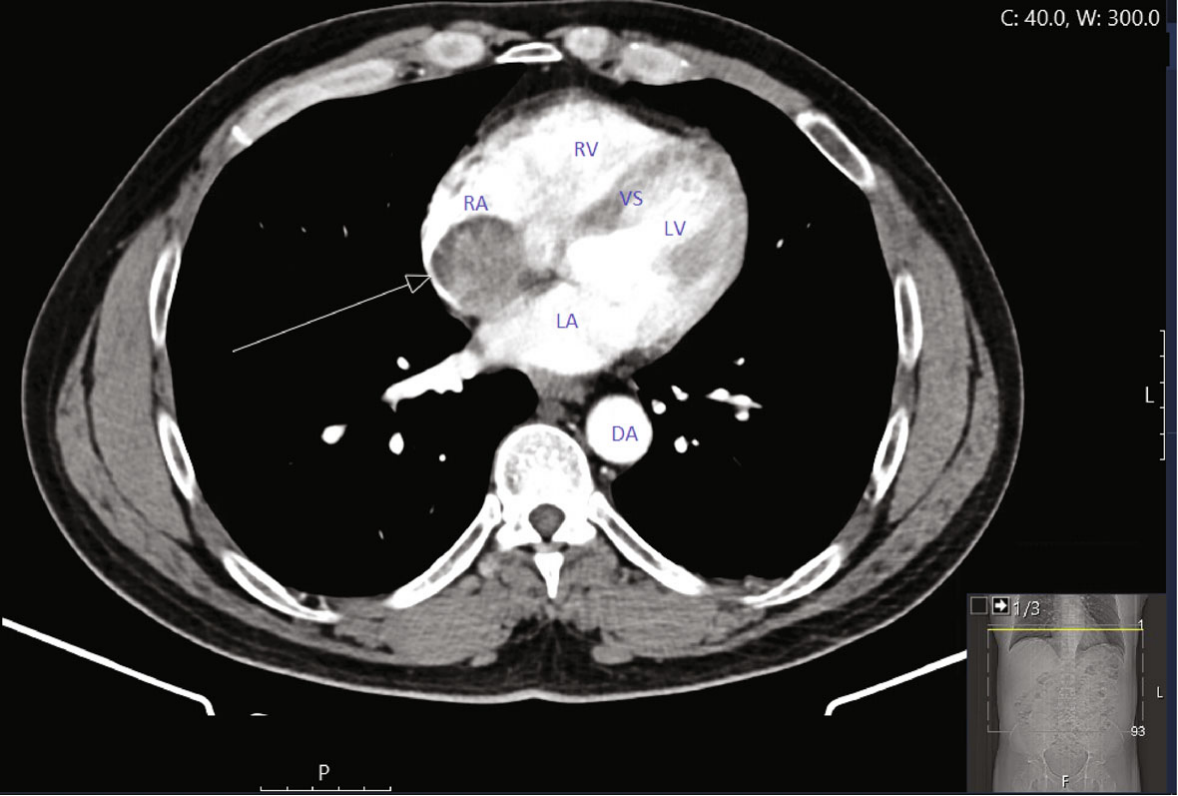
CT abdomen axial view with arterial contrast. Arrow pointing to the right atrial mass. RA = right atrium; RV = right ventricle; VS = interventricular septum; LV = left ventricle; LA = left atrium; DA = descending aorta.

The CT abdomen report detailed that the right atrial mass measured 38 by 35 mm with a tail extending down into the supradiaphragmatic inferior vena cava (Figure 1). Additionally, there was a round low density segment two liver lesion with minimal peripheral enhancement with contrast, possibly a haemangioma.

At this stage, the patient was referred for cardiology assessment. Further questioning revealed symptoms of intermittent chest pain. He did not exhibit any symptoms or signs of heart failure. He had no history of malignancy and no B symptoms. There was no personal or familial history of cardiac disorders. Physical exam and ECG were unremarkable.

**Figure 2:**
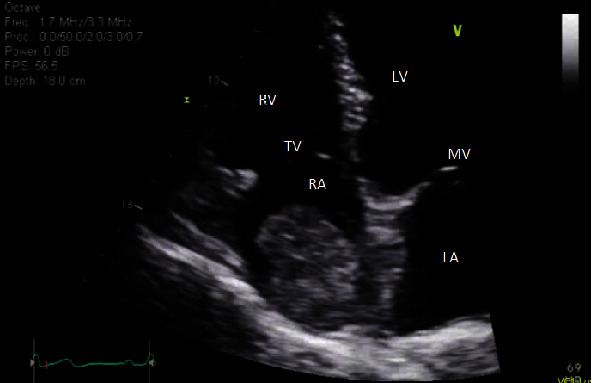
Transthoracic echocardiogram, four chamber view. Mass depicted in the right atrium, not obstructing the tricuspid valve. RV = right ventricle; LV = left ventricle; RA = right atrium; LA = left atrium; MV = mitral valve; TV = tricuspid valve.

Firstly, a transthoracic echocardiogram was performed. This depicted a large echodense mass measuring 4.5 by 3 cm in the right atrium that appeared to arise from the Eustachian valve (Figure 2). It did not seem to originate from the inferior vena cava and was not obstructing the tricuspid valve. The patient had an otherwise normal echocardiograph with left ventricular ejection fraction 55-60%, normal right ventricular function, and no valvular pathology.

**Figure 3:**
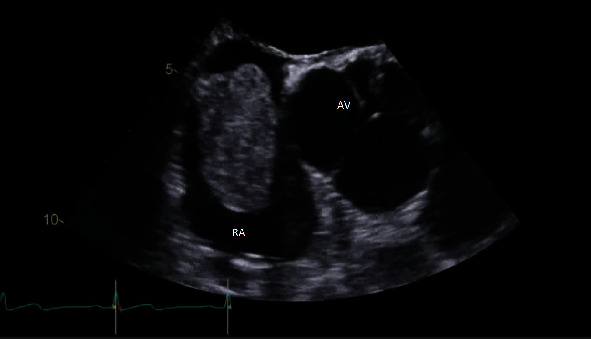
Transoesophageal echocardiogram mid oesophageal aortic valve short axis view demonstrating an echogenic mass in the right atrium (RA) and aortic valve (AV).

Transoesophageal echocardiogram confirmed the above findings and clearly demonstrated the mass, which appeared to have morphological features consistent with myxoma (Figure 3).

**Figure 4:**
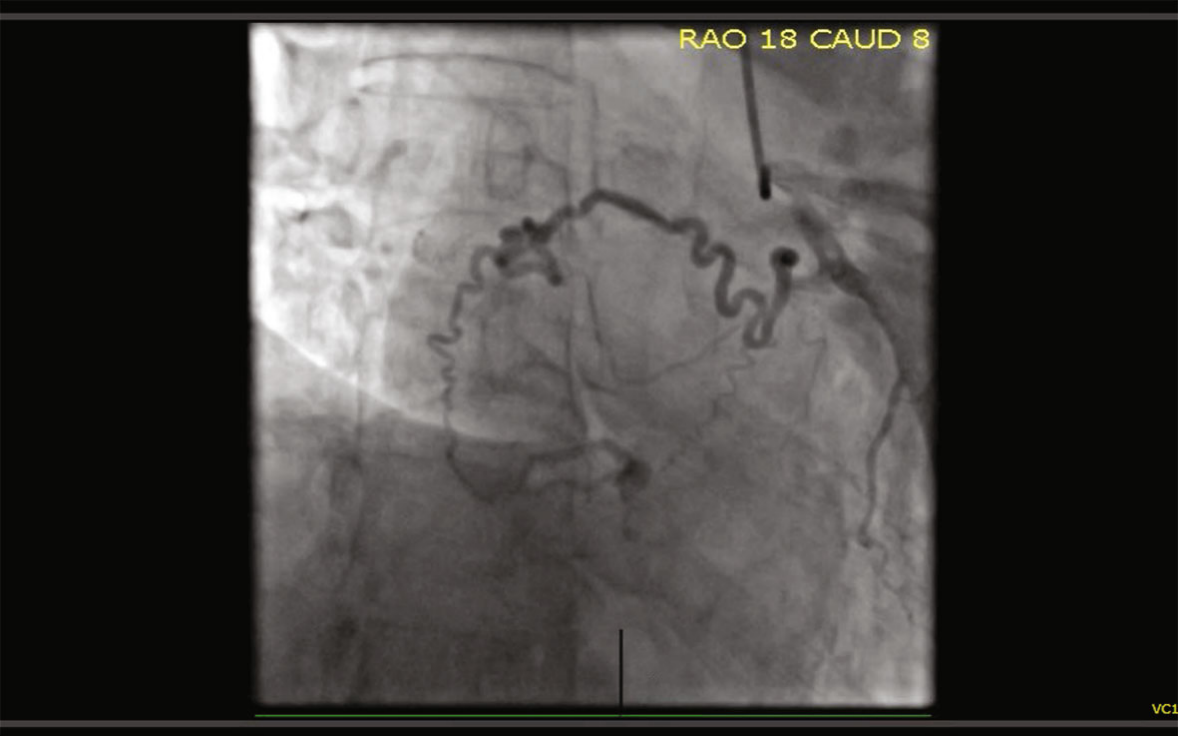
Coronary angiogram RAO caudal view defining the vascular nature of the right atrial mass.

Routine preoperative coronary angiogram was conducted and interestingly revealed large well-developed atrial branches that crossed superiorly over the left atrium and entered the mass in the right atrium. The vessel carried contrast, enhancing the outer layers of the mass (Figure 4).

**Figure 5:**
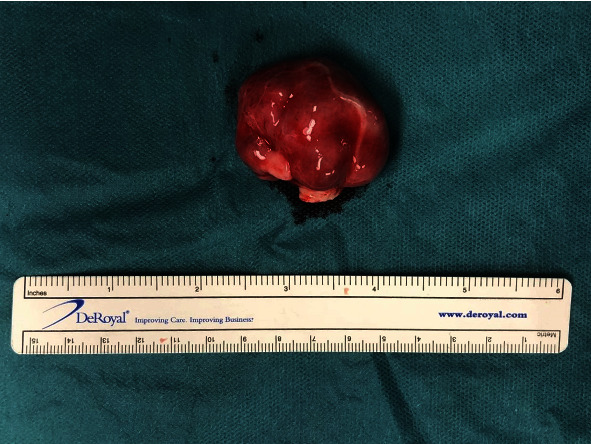
Operative specimen: right atrial mass.

The patient was referred for surgical removal of the right atrial mass. Intraoperative epicardial ultrasound was initially used to interrogate the inferior vena cava to ensure that bicaval cannulation was feasible. It was confirmed that the mass did not encroach into the inferior vena cava and therefore aortic and bicaval cannulation was used to place the patient on cardiopulmonary bypass. The mass was located at the inferolateral aspect of the right atrium and had a fleshy, encapsulated appearance with a sessile stalk. The mass was completely excised (Figure 5). The resulting atrial defect was closed in two layers with 4-0 Prolene.

The patient had an uneventful postoperative period and was discharged five days after his surgery.

**Figure 6:**
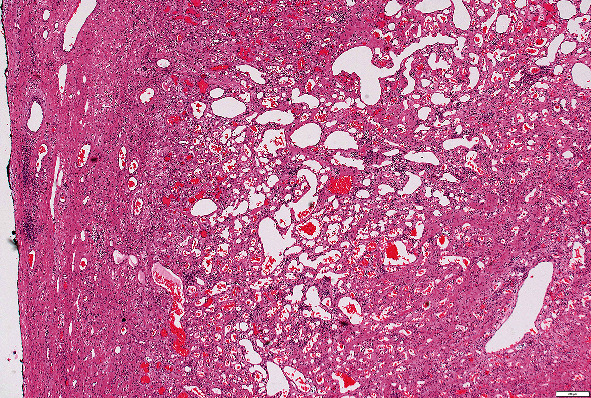
Histological photograph 40x (haemotoxylin and eosin stain). Free surface of the lesion (not attached to the wall of the atrium) to the left; occupying most of the image is a proliferation of thin-walled, differently sized but invariably dilated, capillaries lined by banal endothelial cells.

Histology of the mass demonstrated a cavernous haemangioma composed of dilated vascular spaces lined by endothelial cells (Figure 6). There was no evidence of atypia or malignancy.

Echocardiogram performed four months after his operation depicted a small atrial shunt seen on colour Doppler. There was no residual mass in the right atrium.

## 3. Discussion

Cardiac cavernous haemangiomas are exceedingly rare benign tumours, representing 5% of this group [1, 2]. They are most frequently found in the right side of the heart [2]. These haemangiomas are often asymptomatic and found incidentally during investigation of other diseases [3].

Cardiac haemangiomas are most commonly seen in young adults, with a median presenting age of 43 years, similar to this case [2]. Presentation is likely to occur earlier if the haemangioma causes arrhythmia or heart failure through intracardiac obstruction or valvular involvement due to their size [1]. Echocardiogram is the gold standard imaging for this condition [4].

Whilst preoperative investigations suggested a myxoma, some features of this presentation were atypical. Firstly, right atrial myxomas only account for 23% of all myxomas [5]. Secondly, in this case, the rich vascularity seen on angiogram may have indicated that this was not a myxoma but a haemangioma. Cardiac MRIs and coronary angiogram are useful in determining the vascular nature of these masses prior to resection [4]. The finding in this case of probable hepatic haemangioma is not unusual—many patients have other haemangiomas including skin, pulmonary, pleural, and hepatic [3, 6]. The presence of this hepatic haemangioma may have also given a clue to the true diagnosis of this mass.

Although atrial myxomas make up the vast majority of cardiac tumours, it is prudent to remember that not all cardiac masses are myxomas, as evidenced by this case. In particular, when atypical features are present, the presumptive preoperative diagnosis of myxoma should be avoided.

## Data Availability

The data are available from Dunedin Hospital records.
